# American Dental Association

**Published:** 1870-08

**Authors:** 


### Editorial.

### AMERICAN DENTAL ASSOCIATION.

The Annual Session of this body was held in Nashville, Tenn., August 2d to the 5th, inclusive. The attendance was quite as good as was anticipated, there being about one hundred members present. There was, of course, not so large a number of members from the North and East, as usual, but the South was well represented.

The meeting was quite as interesting and profitable as any of the preceding ones. A definite advance in the science of the profession was quite apparent. Active, energetic, studious, investigating minds are not content to remain in the old paths, especially while there is so much new territory to be opened up, surveyed, appropriated and utilized. It is interesting and gratifying to note the advances made every year.

There were some excellent papers presented, and the discussion for the most part, were of a very interesting character.

In an Association like this, composed for the most part of representative members of the profession from all parts of the country, the sphere of its action should be extended in as many ways, and as far, as it can operate for the good of the profession. Hitherto, the efforts of the Association have been devoted to the improvement of practice, and the better understanding of the branches upon which the practice is based, by the reading of essays and discussions upon the various topics.

In addition to this, we think, this Association might exert some fostering influence over local Associations, particularly in the way of stimulating such as are feeble and organizing new ones.

For three or four years after its organization, this Association accomplished something in this direction; it appointed its committee on local societies. That committee performed some work—entirely at its own cost however—which resulted in the formation of between twenty and thirty societies. But it is said that the condition of the profession has so much changed since that time, that the necessity for such work does not now exist. In one aspect, perhaps, that is true, but in another it is no more true than formerly. It is patent to every one that scientific knowledge has vastly increased within the last few years, but it has no more than kept pace with the increasing demands of the times, so that progress and further attainments are now as much a necessity as ever, and all the means for its procurement as industriously plied. Association should be as extensive and thorough as possible, and every possible assistance to that end should be exercised.

Another subject to which the attention of this National Association could be directed with profit is the subject of professional education. It is true that at a meeting some few years ago, a committee upon this subject was appointed; that committee made a report upon the college education of our students, suggesting, what seemed to them to be the proper course for the colleges to pursue, especially with reference to the branches taught, and the time requisite. The committee was not continued. The single suggestive report had but little effect upon either the colleges or the profession. The effort was a failure simply because it was not continued. The American Dental Association, when it speakes in earnest will be heard, and regarded too by the colleges and the profession. That our system of professional education is sadly deficient no one will pretend to deny, and it is just as apparent that should the American Dental Association take the matter in hand, much improvement might be effected.

A system of normal class instruction for the profession at large might be instituted with great profit; and such an enterprise should emanate from this Association. A man thoroughly competent to give the best clinic instruction should be selected and sent out by this body, visiting the profession whenever he would be kindly received, and give its members whatever aid possible for their professional improvement. Such a work would be most manifest in its results, and perpetual in its duration. We have not the slightest doubt that were such an enterprise organized, the means for carrying it on would be promptly supplied.

In this as well as in other ways that might be mentioned, the American Dental Association might accomplish great good in addition to that resulting from the present mode of procedure.

